# Cellular and Biochemical Actions of Melatonin which Protect Against Free Radicals: Role in Neurodegenerative Disorders

**DOI:** 10.2174/157015908785777201

**Published:** 2008-09

**Authors:** Genaro G Ortiz, Gloria A Benítez-King, Sergio A Rosales-Corral, Fermín P Pacheco-Moisés, Irma E Velázquez-Brizuela

**Affiliations:** 1 Laboratorio de Desarrollo-Envejecimiento, Enfermedades Neurodegenerativas, División de Neurociencias, Centro de Investigación Biomédica de Occidente (CIBO), Instituto Mexicano del Seguro Social, IMSS, Sierra Mojada 800 C.P. 44340 Guadalajara, Jalisco, México; 2 Laboratorio de Neurofarmacología, Instituto Nacional de Psiquiatría, SSA. México, D.F.; 3 Departamento de Química, Centro Universitario de Ciencias Exactas e Ingenierías (CUCEI), Universidad de Guadalajara, Guadalajara, Jalisco. México

**Keywords:** Melatonin, alzheimer, parkinson, oxidative stress, NO, neurodegeneration.

## Abstract

Molecular oxygen is toxic for anaerobic organisms but it is also obvious that oxygen is poisonous to aerobic organisms as well, since oxygen plays an essential role for inducing molecular damage. Molecular oxygen is a triplet radical in its ground-stage (.O-O.) and has two unpaired electrons that can undergoes consecutive reductions of one electron and generates other more reactive forms of oxygen known as free radicals and reactive oxygen species. These reactants (including superoxide radicals, hydroxyl radicals) possess variable degrees of toxicity.

Nitric oxide (NO•) contains one unpaired electron and is, therefore, a radical. NO• is generated in biological tissues by specific nitric oxide synthases and acts as an important biological signal. Excessive nitric oxide production, under pathological conditions, leads to detrimental effects of this molecule on tissues, which can be attributed to its diffusion-limited reaction with superoxide to form the powerful and toxic oxidant, peroxynitrite.

Reactive oxygen and nitrogen species are molecular “renegades”; these highly unstable products tend to react rapidly with adjacent molecules, donating, abstracting, or even sharing their outer orbital electron(s). This reaction not only changes the target molecule, but often passes the unpaired electron along to the target, generating a second free radical, which can then go on to react with a new target amplifying their effects.

This review describes the mechanisms of oxidative damage and its relationship with the most highly studied neurodegenerative diseases and the roles of melatonin as free radical scavenger and neurocytoskeletal protector.

## INTRODUCTION

Free radicals (pro-oxidants) are highly reactive, unstable molecules that have an unpaired electron in their outer shell. They react with several cellular components including nucleic acids, proteins, fatty acids, complex lipids, carbohydrates, etc. [41]. Reactive oxygen (ROS) and nitrogen (RNS) species are formed during normal metabolic activity in a variety of biochemical reactions and cellular function. Their beneficial effects occur at low concentrations and involve physiological roles in cellular signaling systems, induction of a mitogenic response, and cellular responses against infectious agents [125]. Under physiological conditions, the steady-state formation of ROS and RNS is normally balanced by a similar rate of consumption by antioxidants. Oxidative stress results from an imbalance between formation and neutralization of free radicals. Pathologic processes, such as inflammation, ischemia, cancer, neurodegenerative disorders, etc. disrupt this balance by increasing the formation of free radicals in proportion to the available antioxidants. The reactions between cellular components and free radicals lead to immediate damage or death of cells in various tissues, including the central nervous system (CNS) [4, 45].

Examples of free radicals are hydrogen peroxide, hydroxyl radical, nitric oxide, superoxide anion and peroxyl radical. Superoxide is generated *via* several cellular oxidase systems. Once formed, it participates in several reactions yielding various reactive products such as hydrogen peroxide, peroxynitrite, etc. In turn, these can lead to chain reaction byproducts that also act to damage cells (e.g., lipid peroxidation products). An example of a very potent reactant is peroxynitrite which is 1,000 times more potent as an oxidizing compound than hydrogen peroxide [124, 25].

The whole nervous system is rich in metals, particularly, the brain is a specialized organ that accumulates iron ions and it is specially susceptible to oxidative damage since has a high metabolic activity and high content of unsaturated fatty acid [22, 45]. The high level of brain iron may be essential, particularly during development, but its presence also means that injury to brain cells may release iron ions that can lead to oxidative stress *via* the iron-catalyzed formation of reactive oxygen species [53, 65].

Neurodegenerative diseases are a heterogeneous group of disorders characterized by the gradually progressive and irreversible destruction of specific neuronal populations. That loss of anatomically or physiologically related neuronal systems is complex and multifactorial. Although the etiology of the major neurodegenerative (including Parkinson's disease, Alzheimer's disease, Huntington’s disease and amyotrophic lateral sclerosis) is unknown, there is substantial evidence that oxidative stress is a common critical factor in these diseases [63, 77].

Antioxidant properties of melatonin and its metabolites [47, 68, 69, 123] are connected with its neuroprotective activity in several degenerative disorders. The efficacy of melatonin in the inhibition of the oxidative stress has been estimated in various neurodegenerative disorders such as Alzheimer's or Parkinson's disease [3, 62, 104, 111]. Melatonin has a clinical potential for the treatment of neurodegenerative disorders in the central as well as peripheral nervous system [92-103].

## LIFE, BEFORE AND AFTER OXYGEN

The relationship between changes in atmospheric oxygen levels and the evolution of life on the earth are well documented biologically [2, 60]. There was a delayed of more than a billion years between the origin of cyanobacteria and the moment at which the levels of oxygen began to accumulate in the atmosphere [8, 16, 120]. This delay was due to the high presence of dissolved ferrous ion in the oceans, which reacted with free oxygen forming large ferrous oxide deposits. Until then, the only existing organisms were anaerobic cells. The gradual accumulation of oxygen during evolution of organisms that underwent photosynthesis provided the necessary elements for subsequent evolution of aerobic organisms (1,500 million years ago) and must have represented a cataclysm in the history of life [56]. Using oxygen for the generation of energy, aerobic cells obtained more energy from reduced substrates like glucose, due to the complete oxidation of the substrate to CO_2_. These reactions are summarized as follows [2].

(Anaerobic metabolism) glucose → 2 lactate + 56 Kcal/mol glucose

(Aerobic metabolism) glucose + 6O_2 _→ 6CO_2_ + 6H_2_O + 686 Kcal/mol glucose

Because of this, aerobic life forms had an advantage over anaerobes. Anaerobic organisms were also at a disadvantage because the extra oxygen was toxic to them. Oxygen metabolites are toxic to all life forms. For instance, obligated anaerobic organisms develop only in the absence of oxygen. Oxygen toxicity raises several questions: why it is toxic? Why can aerobic organisms prosper in an oxygen-containing atmosphere? Oxygen, in its fundamental state is not toxic, nevertheless, due to its electronic structure, which is includes two unpaired electrons. There are restrictions on its reactivity as an electron acceptor. It has been proposed that reaction of O_2_ + 4e + 4H^+^ → 2H_2_O includes 4 univalent steps (only one electron is transferred): 

O_2_ + 1e → O_2_^–•^

O_2_^–•^+ 1e + 2H^+^→ H_2_O_2_

H_2_O_2_ + 1e + H^+^→ H_3_O_2_ → H_2_O + HO^•^ 

HO^•^ + 1e + H^+^→ H_2_O

Net: O_2_ + 4e + 4H^+^ → 2H_2_O

If oxygen reacts in this manner (and probably it does), the production of superoxide anion (O_2_^–•^) and hydroxyl radical (HO^•^) are intermediates; this represents a major problem for organisms, since these intermediates are potent oxidizing agents. HO^•^ is the strongest oxidizing agent known; this radical can be formed in the following reaction:

H_2_O_2_ + O_2_^–•^ → O_2_ + OH^–^ + HO^•^

Each oxygen species represents a potential threat for cells due to the damage that each can cause to all biomolecules, especially proteins and lipids. In other words, oxygen toxicity is due to the inherent toxicity of the reactive species, either free radicals or other metabolites formed from it [57].

## ENZYME SYSTEMS AND FREE RADICAL INJURY

Accumulative brain injury induced by free radicals is a factor in aging and neurodegenerative diseases [17, 46, 48, 49]. Superoxide anion, O_2_^–•^ is enzymatically produced by the action of cytochrome oxidase, NADH oxido-reductase, xanthine oxidase and phagocytic oxidases. In the latter case O_2_^–•^ is use to eliminate bacteria or tumor cells [29, 66] while O_2_^–•^ is toxic by itself, it can be converted to reactants that are even more reactive.

Hydrogen peroxide (H_2_O_2_) is formed within the cells by the interaction of two O_2_^–•^ in the presence of superoxide dismutase (SOD). Isoforms of SOD in mitochondria and cytosol have been described. Also, H_2_O_2_ is formed by two electron reduction of O_2_.

For the next step either a ferrous or cuprous ion are required in order to produce the anion OH^–^, which is non-toxic, plus the radical HO^•^, which is highly toxic. Cationic iron is the transition metal most often available in this reaction. The HO^•^ reacts easily and indiscriminately with any molecule near to it, and causes damage and production of secondary free radicals. It reacts at a diffusion-controlled rate, either by adding or absorbing a hydrogen atom from other molecules [128].

Free radical damage to macromolecules such as DNA, proteins, and polyunsaturated fatty acids (PUFA) probably produces the most notable functional cellular deficits [48, 122]. PUFA are particularly susceptible to free radicals and once the damage has begun, the reaction is self propagating. In theory, all the lipids in the organism could be oxidized. Initially, free radicals attack methylenic groups adjacent or between ethylenic bonds. Secondly, a chain reaction takes place leading to lipid radicals, alkoxylic and peroxyl radicals. The final products are non-radicals such as alkenes and carbonyl components. Some of these final products are detectable and useful in order to measure the level of lipid peroxidation in a procedure that involves its reactivity with thiobarbituric acid. The peroxyl radical (ROO^•^) formed during the propagation phase is toxic enough to restart the peroxidation of another PUFA[93].

Products of lipid peroxidation can achieve access to the nucleus and harm DNA, besides changing the physiology and structure of the cellular membrane. These changes may be involved in cancer initiation among other pathological processes [21]. Also, cytoskeletal organization is damaged by free radicals. This important cellular structure plays a key role in neuronal physiology and is damaged in neurodegenerative diseases. Free radicals cause a cytoskeletal to collapse around the nucleus. H_2_O_2_ produces neurocytoskeletal damage similar to that found in neurodegenerative diseases. Moreover, free radicals produce neurite damage, and neuronal loss [48, 87] and microtubule network is disrupted in cortical neurons exposed to high levels of free radicals [48]. Additionally, cortical neurons incubated with H_2_O_2 _show distribution changes in beta-tubulin and the 2  isoforms of microtubule associated protein (MAP2) and an abnormal microtubule organization [122]. Moreover, cytoskeletal-dependent functions are affected by free radicals. This notion is supported by the fact that H_2_O_2 _inhibits cytoskeletal dependent processes such as dopamine release by the substantia nigra pars compacta of guinea pig brain [93]. 

## NITRIC OXIDE: SYNTHESIS AND MECHANISMS OF ACTION

Nitric oxide is a multifunctional molecule that participates in processes such as vasodilatation, bronchodilatation, neurotransmission, antimicrobial activity, inhibition of both phagocyte and platelet aggregation [32, 34, 36,40], regulation of cytochrome c oxidase in the mitochondrial respiratory chain [18-20, 30, 32, 112] and mitochondrial biogenesis [79]. As a chemical signal, NO participates through binding and activation of guanylate cyclase and causes smooth muscle relaxation, and a role of NO signaling in response to N-methyl-D-aspartate (NMDA) activated receptors has been described [5]. On the other hand, the mechanism by which tumor cells are eliminated in rodent’s activated macrophages depends on L-arginine [51]; it is hypothesized that macrophages synthesized NO [52] which may be cytostatic and cytolytic if it is produced in higher quantities. Reactive nitrogen species derived from NO such as nitroxyl and peroxynitrite are essential in this process [119].

NO is produced from the enzymatic conversion of the L-arginine mediated by oxide nitric synthase (NOS). The process consumes five electrons and results in formation of L-citrulline and NO, with the participation of electron enzymatic transporters. Such synthesis involves the successive oxidation with the use of NADPH as a transporter of electrons donated by O_2_. NOS contains a reductase and oxygenase domain, with specific recognition sites for flavine mononucleotide (FMN), flavine-adenine dinucleotide (FAD), and nicotinamide-adenine phosphate dinucleotide (NADPH). NADPH, FMN, and FAD transport electrons to heme molecules bound to the oxygenase domain of the enzyme [29, 122]. The electrons flow from one transporter molecule to another, from the reductase domain of the NOS to the heme group, and from the heme to the activated O_2_ (O-O) in order to modify the substrate (L-arginine) that will finally result in L-citrulline and NO production [18].

NOS was originally purified from rat cerebellum [14] and several isoforms were described, although expression of these enzymes is not tissue specific [36, 43, 118]. The endothelial NOS (eNOS, NOS type III) is located in the vascular endothelium and related mainly with the mechanism of smooth muscle relaxation. Neuronal NOS (nNOS, NOS type I) is related with signal transduction in peripheral and central neurons. Both isoforms are calcium-calmodulin dependent. Inducible NOS (iNOS, NOS type II) was isolated originally from murine macrophages; it binds calmodulin but its activity is independent of intracellular calcium. Its expression occurs in response to inflammatory cytokines such as interleukin 1 (IL-1) interleukin 2 (IL-2), tumor necrosis factor alpha (TNF-α) or by lipopolysaccharide S (LPS). Interferon gamma (IFN-γ) acts in a synergistic formin most cases [74].

The difference relative to calmodulin dependence between iNOS and eNOS is that iNOS has strongly coupled domains for the calmodulin structure even with the lack of calcium. eNOS and nNOS require previous formation of calcium-calmodulin complex, followed by the enzymatic engagement, for which prior rises of intracellular calcium are required [36]. 

The NOS in absence of L-arginine works in an uncoupled mode and produces large quantities of O_2_^–•^. NO can react with molecular O_2_ giving highly reactive radicals in addition to nitrites and nitrates, which are easily measurable. Under physiological conditions, O_2_ is not the primary target of NO. The most probable reactions are:

2NO^•^ + O_2_ → 2NO_2_^•^→ NO_2_O_4_

NO_2_O_4_ + H_2_O → HNO_2_ (nitrite) + HNO_3_ (nitrate)

NO^•^ + H_2_O → NO_2_O_3_

NO_2_O_3_ + H_2_O → HNO_2_

NO also reacts with O_2_^–•^ initiately, yielding NOO^–^ and eventually HO^•^, both of which are highly reactive; moreover, intermediate products are powerful inducers of lipid, protein, and DNA peroxidation; the consequences of these reactions are analyzed later [136].

NO^•^ + O_2_^–•^→ OONO^•^ → OONOH → NO_2_ + HO^•^

With transition metals, especially heme iron, NO binds to the heme moiety of guanylyl cyclase and activates it to form cGMP. When cyclic oxygenase binds to the heme group the production of prostaglandin is increased; as a result, other enzymes that contain iron within a heme group are targets of NO. Catalase, cytochrome C, hemoglobin and peroxidase are examples:

NO^•^ + X-Fe^x^→ (X- Fe^x^ - NO^•^) →  X-Fe^x-1^- NO^+^ (nitrosum ion)

NO^•^ + Y-Fe^y^→  (Y- Fe^y^ - NO^•^) →  Y-Fe^y-1^- NO^-^ (nitroxidum ion)

NO^•^ + Hb(Fe^2+^)O_2_→  Hb (Fe^3+^) + NO_3_^-^

DNA damage induced by NO or ONOO^-^ activates poly-ADP ribose synthetase (PARS) which coordinates DNA repair through the addition of ADP-ribose and the regulation of histones, high mobility proteins (HMGPs), nuclear matrix proteins (NMPs), topoisomerase I and the Ca^++^-Mg^++^-dependent endonuclease. When NO inhibits ribonucleotide reductase (RR) the delivery of deoxyribonucleotide triphosphate is decreased (NTP→dNTP). The prolonged repair of DNA increases the activation of PARS. At the same time, constitutive poly-ADP ribose glycohydrolase degrades poly-ADP ribose. Four ATP molecules are required to rebuild nicotineadenine-diphosphate (NADP) from nicotinamide. Glycohydrolase from poly-ADP-ribose and PARS initiates a vicious cycle which reduces the levels of NAD, cellular energy and, ultimately, produces cell death [43, 137]. Because of these findings, NO is often considered as a dangerous molecule more than an essential element in neurophysiology [7,76].

Because NO has an important role in neurotransmission it is important to consider the mechanisms involved in its synthesis. NO production requires the engagement of calcium-calmodulin complex for the activation of constitutive synthases in neurons and endothelial cells (nNOS and eNOS) [36]. This is a very refined mechanism, with the amounts of NO being limited and precise. The production of NO in intact neurons occurs in response to excitatory stimuli that required Ca^++ ^[38]. According to the location of the enzyme, activation of NOS is coupled to: a), stimulation of postsynaptic receptors by neurotransmitters leading to the mobilization of calcium, and, b), action potentials in the presynaptic terminals that induce calcium flows through voltage sensible channels [105, 136].

The activation of glutamate receptors is the main postsynaptic stimulus for NO synthesis. NMDA receptors associated with ionic channels have a high permeability to calcium [39]; although other ionic channels associated with glutamate receptors like alpha amino-3-hydroxy-5-methyl-4 isoxazolpropionate (AMPA) and kainate sensitive receptors [81], have also been implicated [126]. Other neuromodulators or neurotransmitters possibly related with NO synthesis are serotonin, bradykinin, acetylcholine, and noradrenaline [91]. Even though neuronal and endothelial synthases are considered constitutive, these molecules can also be induced by particular events such as neuronal plasticity, development, stress and direct lesions. Rather few neurons have been detected that expressed messenger RNA (mRNA) for NOS in lumbar ganglia of normal rats. Nevertheless, increased levels of NOS mRNA were found in one third of the neurons two days after the transection of the sciatic nerve, and this rise was maintained for at least two months [129].

An increase in the levels of NOS mRNA has also been detected in response to stress [24], lactation [27], in the pituitary gland in response to gonadectomy and in the hippocampus as result of Alzheimer’s therapy with Tacrine^™^ and lithium [6].

In the pineal gland the production of NOS is regulated by a physiological stimulus. After 8 days of constant light exposure (which reduces melatonin synthesis), the activity of NOS decreases by 80% and the normal activity is restored by normal light/dark cycles for two days. Noradrenaline appears responsible for this photoneural regulation [106].

A problem with NO occurs when it is produced in large quantities. iNOS can produce high amounts of NO for long periods. The iNOS route therefore represents a response element of the cytotoxic cellular immune response, and the induction of iNOS results in 30-fold increase in NO formation in the CNS [122].

Macrophages, smooth muscle cells, and endothelial cells express iNOS when induced by proinflammatory agents such as LPS, IL-1β and/or TNF-α. Structurally, iNOS is strongly bounded to calmodulin; therefore, calcium-calmodulin complex is not required for activation. The main step in the synthesis of NO is the cellular concentration of the enzyme; its signaling pathway involves protein-kinase C [114] and induces the activation of the iNOS gene. ADP-ribosylation represents a crucial point in this signaling pathway [50]. It has been observed that the ADP-ribosylation inhibitors such as nicotinamide and benzimidine prevent the induction of NOS activity by IFN-γ and LPS in macrophages. Once iNOS has been activated, the synthesis of NO from L-arginine can reach a rate of 100nmol/h per mg of macrophage activated protein; this rate can be maintained for a long period of time. The regulation is at the transcriptional level [18, 108].

The properties of NO are peculiar. For example, though it is a gas (in paranasal sinus and lungs, it is maintained as such) it stays dissolved in solution as a non-electrolytic substance capable of spreading to any compartment, since it is both liposoluble and hydrosoluble. NO at 1 nM is sufficient to interact with a billion synapses [81]. The half life of the NO is very short (5 seconds), but its diffusion is extremely rapid. Yet at 20 µm beyond its production site, the concentration is reduced to 10%. In spite of such a short life, NO generated at a given point can influence a radius of approximately 0.3 mm (the extent of the synaptic groove is about of 20 nm). Moreover, NO affects neuronal structure. It induces neurite formation and dendrite branching in neuronal cells [134]. During development it also orchestrates neurite outgrowth and filopodial dynamics, cell migration of enteric neurons, glial migration and axonogenesis of pioneer fibers and it may regulate cell motility in the developing and regenerating vertebrate nervous system [13]. In addition, evidence has suggested that dementias are cytoskeletal disorders that involve loss of axons and dendrites of neurons in the CNS and consequently disruption of synaptic connectivity [11]. 

With respect to mitochondrial cytochrome c oxidase regulation, NO binds reversibly to the binuclear oxygen binding site in cytochrome c oxidase (complex IV) in competition with oxygen [19, 31, 75], this results in rapid inhibition of respiration in whole mitochondria and in the presence of physiological levels of O_2_. The mechanisms involved are complex, with greater inhibition occurring as the O_2_ concentration decreases, through a mechanism that includes regulation by the mitochondrial inner membrane [20, 30, 112]. Interestingly, mitochondria has one NOS isoform (nNOS modified posttranslational), it is consistent with direct regulation of mitochondrial energy production by NO [33]. Higher NO levels disrupt the respiratory chain and may cause changes in mitochondrial calcium flux [133]. It was suggested that NO is probably the primary agent involved in preferential complex I inhibition following acute glutathione depletion in dopaminergic neurons in PD [54,107]. In contrast, other RNS, such as N_2_O_3_ and ONOO^-^ alter mitochondrial function through the irreversible modification of proteins. For instance, ONOO^-^ induces inhibition of complex II, inhibition of the ATP synthase and nitration of Mn superoxide dismutase [26, 67, 89]. In addition, radical nitroxyl (HNO) inhibits mitochondrial respiration through the inhibition of complexes I (NADH oxido-reductase) and II (succinate dehydrogenase), most probably *via* modification of specific cysteine residues in the proteins [113].

## FREE RADICALS AND NEURODEGENERATIVE DISEASES

Recent evidence has focused attention on the role of oxidative stress in various acute and chronic neurodegenerative diseases [87]. An increasing number of physicians are also recommending antioxidant therapies, such as high doses of vitamin E, for subjects with AD and other neurodegenerative disorders. Vitamin E, *Ginkgo biloba*, and selegiline are three putative antioxidants that have been tested in randomized multicenter trials in the US [35].

Oxidative insults, whether over-excitation, excessive release of glutamate or ATP depletion caused by stroke, ischemia or inflammation, exposure to ionizing radiation, heavy-metal ions or oxidized lipoproteins may initiate various signaling cascades leading to apoptotic cell death and neurodegenerative disorders [121]. Pathophysiologic processes common to both vascular (multi-infarct) dementia and dementia of the Alzheimer’s type may include microglial activation with the resultant generation of inflammatory cytokines and neurotoxic free radicals, decreased secretion of nerve growth factor by astrocytes, excess release of glutamate with associated neurotoxicity, and loss of cholinergic neurons [73].

**Fig. (1):**
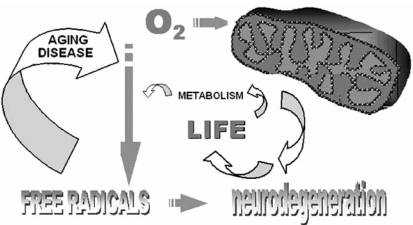
Oxygen and the continuity of life through the most suitable energy production (right). On the left side: metabolism, ageing and pathological conditions involving free radicals and oxidative stress.

Mitochondria are intimately involved in the production of ROS through one-electron carriers in the respiratory chain (see Fig. **1**); mitochondrial structures are also very susceptible to oxidative stress, as evidenced by massive induction of lipid peroxidation, protein oxidation, and mitochondrial DNA (mtDNA) mutations [64]. Oxidative stress can induce apoptotic death and mitochondria have a central role in this and other types of apoptosis, since cytochrome c release in the cytoplasm and opening of the permeability transition pore are important events into the apoptotic cascade. Mutations in mtDNA have profound implications. Maternal inheritance of mtDNA is basic for hereditary mitochondrial cytopathies; the accumulation of somatic mutations of mtDNA with age the basis of mitochondrial theory of aging, which includes a vicious cycle of mtDNA damage, altered oxidative phosphorylation and overproduction of ROS [61].

The efficiency of the mitochondrial electron transport chain (ETC) is reduced in multiple tissues, including brain, from patients with Parkinson's disease (PD) and Alzheimer's disease (AD). The ETC defects are specific to each illness, e.g., complex I in PD and complex IV in AD. In mtDNA-deficient clonal neuronal cells hybridized with mtDNA (“cybrids”) from PD or AD patients these defects are transferable with mtDNA and lead to increased production of ROS [117]. ETC inhibition *in vivo* increases production of the toxic OH^•^, but the underlying mechanisms vary as a function of which ETC complex is inhibited. 

There exists the possibility that tryptamine-4,5-dione (T-4,5-D) and perhaps other putative intraneuronal metabolites formed by the O_2_^-^-/H_2_O_2_/oxo-iron-mediated oxidations of 5-hydroxytryptamine (5-HT, serotonin) might be endotoxins that contribute to neurodegeneration in brain regions innervated by serotonergic neurons. These metabolites are caused by methamphetamine (MA), glutamate-mediated oxidative toxicity, ischemia-reperfusion, and other neurodegenerative brain disorders [131].

Also, metals have an important role in neurodegeneration. Iron can contribute to free radical damage by catalyzing the formation of the OH^•^, inducing secondary initiation of lipid peroxidation and by promoting the oxidation of proteins. The iron chelator, deferoxamine, can limit these oxidative reactions and it scavenges peroxynitrite independent of iron chelation [86]. The increase in brain iron associated with several neurodegenerative diseases may lead to an increased production of free radicals *via* the Fenton reaction. The intracellular iron is usually tightly regulated, being bound by ferritin in an insoluble ferrihydrite core. The neurotoxin 6- hydroxydopamine (6-OHDA) releases iron from the ferritin core by reducing it to the ferrous form [32]. In the presence of ferritin, both 6-OHDA and THB strongly stimulate lipid peroxidation, an effect abolished by the addition of the iron chelator deferoxamine. These results suggest that ferritin iron release contributes to free radical-induced cell damage *in vivo*.

Iron accumulation could be an important contributor to oxidative damage of AD [32]. Redox-active iron is associated with the senile plaques and neurofibrillary tangles, the pathological hallmark lesions of this disease. Iron associated with the lesion induces *in situ* oxidation and readily catalyzes an H_2_O_2_-dependent oxidation. With deferoxamine the iron can be re-bound to the lesions. Characterization of the iron-binding site suggests that binding is dependent on available histidine residues and on protein conformation.

In relation to free radical membrane damage and neurotoxicity in AD, an emerging hypothesis contends that β-amyloid toxicity results from peptide-mediated free radical reactions and the generation of ROS. Recently, it has been reported that reactivity of β-amyloid toward the oxidation-sensitive enzyme glutamine synthetase is related to the peptide's reactivity toward the spin trap phenyl-tert-butyl nitrone (PBN) and the neuronal damage may be due, in part, to oxidative processes initiated by amyloid-derived free radicals species. Electron paramagnetic resonance (EPR) spin labeling techniques and spectrophotometric assays provide evidence that a portion of synthetic β-amyloid [26-36] demonstrates hydrogen peroxide-like reactivity toward Fe^2+^, nitroxide spin probes, and membrane proteins of neocortical synaptosomes [23].

Aluminum may facilitate increases in intracellular Ca^2+^ and ROS, and potentially contributes to neurotoxicity induced by other neurotoxins [127]. Although its mechanism of action is unknown, aluminum alters Ca^2+ ^flux and homeostasis, and facilitates peroxidation of membrane lipids. Since both abnormal increases of intracellular Ca^2+^ and oxygen free radicals are present in pathways leading to neurodegeneration, the effect of aluminum on these parameters was examined *in vitro* using primary cultures of cerebellar granule cells. Exposure to glutamate (1-300 µM) caused a concentration-dependent uptake of ^45^Ca in granule cells to a maximum of 280% of basal value. Pretreatment with AlCl_3_ (1-1000 µM) has no effect on ^45^Ca accumulation, but increased the uptake induced by glutamate. Similarly, AlCl_3 _has no effect on intracellular free Ca^2+^ levels measured using the fluorescent probe fura-2, but it potentated the increase induced by glutamate. The production of ROS was examined using the fluorescent probe dichlorofluorescin. By itself, AlCl_3_ had little effect on ROS production; however, AlCl_3_ pretreatment increased ROS production induced by 50 µM Fe^2+^ [78]. Such mechanisms may be involved in the progression of neurodegenerative diseases, including AD and amyotrophic lateral sclerosis (ALS).

AD and Familial ALS may be linked through a common mechanism. In Familial ALS, SOD-Cu(I) complexes are affected by H_2_O_2_ resulting in free radical production; in AD, the reduction of Cu(II) to Cu(I) by APP involves an electron-transfer reaction and could lead to a production of OH^•^, thus, copper-mediated toxicity of APP-Cu(II)/(I) complexes may contribute to neurodegeneration in AD[78].

NO is involved in acquired immune deficiency syndrome (AIDS) dementia complex and viral encephalitis [58]. It has also been demonstrated increases in iNOS expression in brains of patients with multiple sclerosis [126], in AD [23], AIDS [72]. In AIDS mediated neurodegeneration, HIV, or the glycoprotein 120 (gp120) induces iNOS and, at the same time, releases macrophage proinflammatory cytokines. Cytokines from macrophages or neurons induce iNOS in glial cells. Furthermore, gp120 protein is lethal for cortical neurons in culture; this effect is reduced by NOS inhibitors [59]. Other proteins from VIH virus shield such as gp160, and gp41 induce iNOS in astrocytes and microglial cells cultures. The process is similar to the NMDA agonist receptor mechanism, probably more related with peroxynitrite than NO [58]. Excessive activity of glutamate acting through NMDA receptors mediates cellular death in cerebral focal ischemia [116]. Neurotoxicity of glutamate plays an important role in neurodegenerative diseases such as Huntington and AD. Activation of NMDA receptors for 5 minutes and the subsequent increase in intracellular calcium initiates the damage; if NMDA it is applied during 5 minutes to cortical neuron cells in culture it causes cellular death 24 hours later. This "delayed neurotoxicity” depends not only of ionic calcium influx but also on the influx mechanism, due to calcium efflux through NMDA channels; this is the most toxic method in which to provoke damage. There are some possible mechanisms to protect against delayed neurotoxicity: a) inhibiting NOS; b) removing NO substrates (L-arginine) and c) in the presence of reduced hemoglobin, which is NO scavenger. In knockout mice for nNOS, it is possible to avoid glutamate neurotoxicity, as well as in oxygen-glucose combined deprivation [70]. 

## MELATONIN IN FREE RADICAL SCAVENGING PROCESSES

Evidence exists, as seen previously, for oxidative damage to macromolecules in ALS, Huntington disease, PD, and AD. Potential therapeutic agents, based on the pathophysiology described, include inhibitors of glutamate release, antagonists of excitatory aminoacids, strategies to improve mitochondrial function, trophic factors and free radical scavengers [35, 92].

Melatonin (N-acetyl-5-methoxytryptamine) a molecule produced by a diversity of organisms, from algae to humans, has an evolution parallel to that of aerobic metabolism [98, 100, 135]. In all organisms melatonin is primarily produced during the night; this includes man. Melatonin is synthesized by the pineal gland, retina, gastrointestinal tract, etc, and is secreted by the pineal gland. Nevertheless, nocturnal melatonin production decreases with age, such that in old animals the quantity of melatonin is very limited [92]. Melatonin protects against LPS-mediated toxicity in liver and brain [110, 121]. In animals treated by pentobarbital, there is a decrease in the levels of gluthatione peroxidase activity. Also, melatonin protects against the rise lipoperoxidation products induced by LPS [109, 110]. LPS is used to induce endotoxic shock in experimental animals [29, 66, 119, 130], and it stimulates the production of NO and free radicals, which promotes nuclear and mitochondrial DNA damage [119]. There is evidence showing a quick diffusion of melatonin into the nucleus; this protects DNA from genetic damage induced by LPS [109, 110]. 

Melatonin’s capacity to reduce lipoperoxidation damage of the membrane lipids it is also manifested during the process of ischemia-reperfusion. In a study of these phenomena, high quantities of highly toxic free radicals are generated, including O_2_^•^ and OH^•^. The primary sources of these oxidants include endothelial cells, macrophages, polymorpho-nuclear leucocytes, and hepatic cells. Experimentally, advanced administration of melatonin in animals experiencing ischemia-reperfusion reduces the levels of malondialdehydes (MDA) and 4-hydroxyalkenals (4-HDA), metabolites of membrane-lipid peroxidation, and therefore indicators of injure [135]. The mechanism by which melatonin detoxifies highly reactive oxidants is donating an electron to these electrophylic compounds [73]. Thereafter, the indolyl cation formed by the transfer of one electron in the presence of the O_2_^-•^ becomes the metabolite kynuramine [88].

The protective effects against lipid peroxidation by melatonin has been evaluated extensively [61] utilizing xenobiotic substances such as paraquat and diquat, substances that in presence of p450 NADPH-cytochrome reductase induces the generation of O_2_^-•^ [82, 132, 138]. Melatonin administered to rats previously injected with paraquat or diquat prevents oxidative changes in liver, lungs and kidneys and increases survival of the animals. In addition, in animals treated with LPS, melatonin abolished the increment in lipid peroxidation and managed to offset the hepatic degenerative changes. In experiments *in vivo*, the administration of melatonin prevented carbon tetrachloride (CCl_4_) damage, CCl_4_ is a toxin metabolized by cytochrome p450, to produce trichlorometyl radicals and cause the production of other free radicals which produce lipid peroxidation in the kidney and liver. In other studies, rat brain homogenates were incubated with kainate or hydrogen peroxide resulting in lipid peroxidation, but in the presence of melatonin, there was a reduction in lipid damage. Additionally, melatonin’s effect was in a dose-dependent fashion [61, 83]. There are a number of characteristics which melatonin possess which aids its ability to inhibit lipid peroxidation: a) it is highly lipid soluble [100]; b) It scavenges OH^•^ radicals, which toxic enough to initiate lipid peroxidation [92, 98]; c) it increases the efficacy of other antioxidants as vitamin E [15, 92, 97, 98] and vitamin C [35]; d) it may scavenge peroxyl radicals (ROO^•^) [94, 99] who propagate peroxidation, as well as the highly reactive singlet oxygen [35]; e) it increases the activity of gluthatione peroxidase and superoxide dismutase [35, 92, 95].

In rat brain homogenates in the presence of NO, liberated by sodium nitroprusiate [116] it was possible to protect against the lipid membrane peroxidation with melatonin or vitamin E. The neuroprotective effects of melatonin have also been shown other conditions: a) the oxidative effects of hyperbaric oxygen exposure [84, 131]; b) after the induction of transitory cerebral ischemia and reperfusion in gerbils [44, 86]; c) the combination of diverse effects on antioxidant enzymes, the direct elimination of free radicals, the clear access through all morphological barriers and the penetration to all subcellular compartments, are characteristics that make melatonin potentially important factor in the antioxidant defense system [35, 48, 82, 92, 95, 97]; d) in grastrointestinal lesions in rats induced by ethanol, indomethacin or acetic acid [71, 80]; e) lesions in hippocampus and striatum induced by MPTP; in this case melatonin is proposed as a feasible alternative to the management of PD [1, 32].

It is known that lipid peroxidation disturbs order and the lipid dynamic in biological membranes. After oxidative stress, membrane fluidity, a parameter that reflects activity in the phospholipid bilayer, is decreased [28]. Melatonin preserves efficiently microsomal membranes against the rigidity induced by lipid peroxidation [37]. The importance of these findings resides in the close relation of numerous membrane functions with membrane fluidity, i.e. signal transduction, solute transport, and inactivity of membrane-associated enzymes [42, 115]. Even slight alterations in membrane fluidity are related with aberrant cellular function and neurological disease [135, 139, 140]. Recently, it was shown that pinealectomy, which leads to a drop of melatonin concentrations in the blood, worsen the rigidity in the microsomal membranes due to aging [101]. The ability of melatonin to prevent membrane rigidity mediated by oxidative stress suggests that melatonin may play some cell protective actions because of it stabilizes cellular membranes.

## MELATONIN AS A NEUROCYTOSKELETAL PROTECTOR: IMPORTANCE FOR NEURONAL PLASTICITY AND SYNAPTIC CONNECTIVITY LOST IN NEURODEGENERATION

H_2_O_2_ causes loss of neurites and a cytoskeletal retraction toward the perinuclear region. Melatonin prevents microfilament and microtubule collapse in N1E-115 cells as well as the increased lipid peroxidation and apoptosis caused by H_2_O_2_. Our findings also indicate that melatonin restores neurite formation, microtubule enlargement, and microfilament organization in microspikes and growth cones in cells cultured with H_2_O_2_. While, the PKC agonist caused cytoskeletal reorganization in the presence of H_2_O_2_, the PKC inhibitor, bis-indolylmaleimide, blocked neurite formation and cytoskeletal reorganization elicited by melatonin. In addition, the CaM antagonist, ophiobolin, was not able to protect the cells against the damage caused by H_2_O_2. _However, PMA and ophiobolin resembled the melatonin effects in cells treated with H_2_O_2_ and a cytoskeleton organized in neurites and a network all over the cytoplasm was observed. By contrast, the melatonin receptor antagonist did not abolish the protective effects of melatonin against the damage caused by H_2_O_2_. Our data suggest that melatonin can be a potential therapeutic agent in the treatment of neurodegenerative diseases through prevention of the cytoskeletal damage caused by free radicals and by restoration of cytoskeletal organization and neurite formation.

Since melatonin levels often are decreased in psychiatric illnesses, and as we described here the indolamine elicits neurite formation, it is plausible that the low levels of melatonin found in patients with schizophrenia and depression affect neurite formation and therefore neurodevelopment in these individuals. Furthermore, abnormalities in the process of neurite outgrowth could explain increased pruning found in patients with schizophrenia as abnormally formed neurites could be prone to higher rates of removal. Moreover, disconnectivity between brain structures, which has been proposed as the anatomical basis for psychosis could result from defects in neurite formation associated with low levels of melatonin. These data suggest that melatonin could have utility in the treatment of schizophrenia but this needs further study [11].

Melatonin, the main product synthesized by the pineal gland has important properties that make this compound useful in the treatment of dementia [85, 96, 102]. This indole is a potent free radical scavenger and also it governs the assembly of the three main cytoskeletal components stimulating neuritogenesis [11]. In N1E-115 cells, melatonin increases neurite formation and in this process participate the selective activation of the alpha isoform of PKC. Activation of PKC alpha is followed by its translocation from the cytosol to the membrane cytoskeletal fraction and increased vimentin phosphorylation and vimentin intermediate filament reorganization [9]. Moreover, in MDCK epithelial cells, melatonin through PKC activation increases vinculin phosphorylation and focal adhesion formation, a process that implies cytoskeletal reorganization [90]. Additionally, melatonin antagonism to Ca^2+^/Calmodulin (CaM) decreases the activity and autophosphorylation of CaM kinase II, a key protein kinase, involved in neurite maturation [12], and caused neurite enlargement through an increase in tubulin polymerization caused by its CaM antagonism [55]. Recently, we showed in N1E-115 cells that melatonin preclude microfilament and microtubule collapse as well as the increased lipid peroxidation and apoptosis caused by free radicals generated by hydrogen peroxide (H_2_O_2_) [10]. Moreover, the indole restores neurite formation, microtubule enlargement, and microfilament organization in microspikes and growth cones in cells damaged with H_2_O_2 _through PKC activation. The PKC agonist, phorbol 12-myristate 13-acetate (PMA) caused cytoskeletal reorganization in the presence of H_2_O_2_, while the PKC inhibitor, bisindolylmaleimide, blocked neurite formation and microfilament reorganization elicited by melatonin. Thus, the stimulatory properties of melatonin on neuritogenesis as well as its modulatory actions on cytoskeletal protein phosphorylation suggest that melatonin may reestablish neurite formation and the basal levels of phosphorylated tau in N1E-115 cells treated with okadaic acid. Thus, in the present study, we evaluated the effects of melatonin in neuritogenesis by counting the damage rounded cells as well as cells bearing microspikes and neurites. Also, we measured the amount of phosphorylated levels of tau in N1E-115 cells treated with okadaic acid and melatonin. The results showed that in N1E-115 cells, melatonin reestablished neuritogenesis in okadaic damaged cells and blocked abnormal tau phosphorylation caused by this compound. Data strongly suggest that melatonin may improve cognition by impeding neuronal damage caused by tau hyperphosphorylation and cytoskeletal collapse and through establishing new neuronal pathways by neuritogenesis stimulation.

**Fig. (2):**
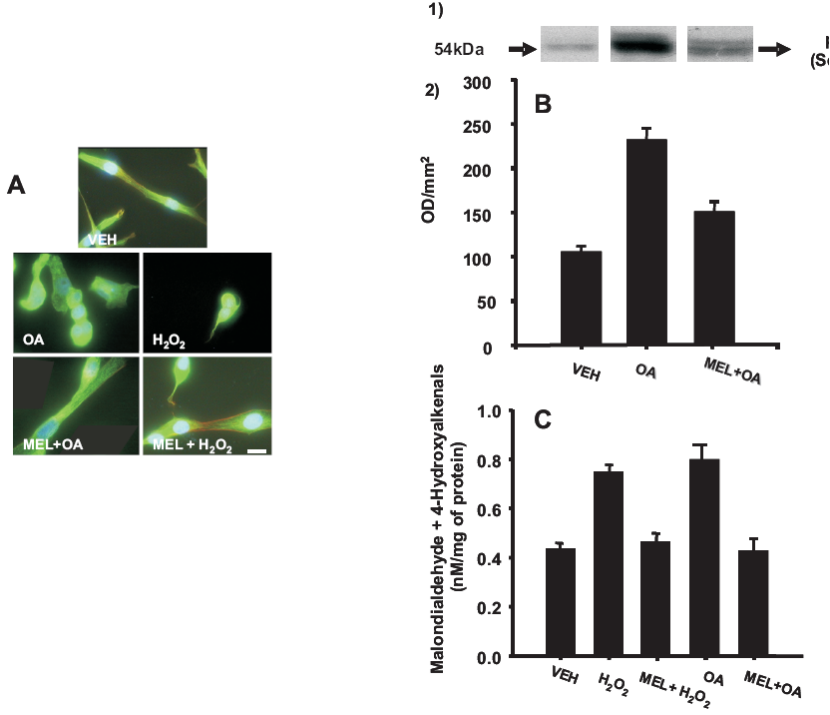
Melatonin effects on cytoskeletal alterations produced by okadaic acid (OA) or hydrogen peroxide (H_2_O_2_) in N1E-115 cells. (**A**) N1E-115 cells were incubated with the vehicle (VEH), 15 nM OA for 24 h (OA), 100 µM H_2_O_2_ for 1 h (H_2_O_2_), 0.1 µM melatonin for 6 h before treatment with 15 nM okadaic acid for 24 h (MEL+AO) or 0.1 µM melatonin for 3 h before 1 h treatment with 100 µM H_2_O_2_ (MEL+ H_2_O_2_) Cultures were fixed and simultaneously stained with an antitubulin antibody (green), RITC-phalloidin (red) and DAPI for detection of microtubules, actin microfilaments and the nucleus, respectively. Bar = 10 mm. (**B**) Melatonin effects on tau hyperphosphorylation induced by OA in N1E-115 cells. 1) Melatonin effects in phospho-tau levels were analyzed by Western blot. Cells were incubated with the VEH, OA and MEL+OA. Representative autoradiogram of p-tau in the VEH, OA, or MEL+OA. 2) Optical density (mm^2^) of p-tau immunoreactivity is showed in the histogram. Results represent the mean ± S.E.M. of three experiments done in quadruplicate. (**C**) Melatonin effect on lipid per-oxidation induced by OA and H_2_O_2_. Neuroblastoma cells were incubated with the VEH, H_2_O_2_, MEL+ H_2_O_2_, OA and MEL+OA. Cells were homogenized and both MDA and 4-HDA were quantified. Results represent the mean ± SEM of three experiments done in duplicate.

To test whether melatonin at the physiological cerebrospinal fluid circulating concentration modified hyperphosphorylation of tau caused by okadaic acid, protein cell homogenates were separated in SDS-PAGE, proteins transferred to nitrocellulose paper and tau identified by Western blot by using an antibody that recognized phosphorylated 404 serine and C-17 antibody that recognized two tau isoforms at carboxyl terminal site. Fig. (**2**) show that okadaic acid treatment increased the phosphorylation of tau at Ser-404. Melatonin added before, simultaneously or after okadaic acid treatment decreased tau hyperphosphorylation caused by this compound respectively (Fig. **2**, **A-B**). Densitometric analysis showed that okadaic acid augmented phosphorylated tau by 121% regarding the vehicle incubated cells. While melatonin decreased the relative quantity of phospho-tau when was added before, simultaneously, or after okadaic acid by 76%, 41%, 81%, respectively (Fig. **2**). No differences were found when tau was recognized by C-17 antibody in cell extracts obtained from cells cultured with okadaic acid, melatonin or combination of melatonin and okadaic acid treatments.

Neuroprotective actions of melatonin have been shown to occur through its intracellular antioxidant mechanisms and its neurocytoskeletal protective effects. Previously, we showed in N1E-115 cells damaged with H_2_O_2_ that melatonin restored neurite formation, microtubule enlargement, and microfilament organization in microspikes and growth cones. Hyperphosphorylation of tau has been shown to occur associated with high levels of oxidative stress and considered as an important hallmark of most neurodegenerative diseases. Moreover, we observed in N1E-115 cells that melatonin prevented microtubule disruption as well as increased lipid peroxidation and apoptosis caused by the phosphatase inhibitor okadaic acid (OA), a specific inhibitor of the serine/ threonine proteins phosphatases 1 and 2A that induces molecular and structural changes similar to those found in Alzheimer’s disease. It is known that tau protein plays a key role in microtubule stabilization and in neurite formation and that dynamic change in microfilament organization also occurs during neurite formation. Therefore, in this work we evaluated the effects of melatonin on neuritogenesis and tau phosphorylation in N1E-115 cells incubated for 24 h with 15 nM OA in the presence of 10^-11^, 10^-7 ^or 10^-5^ M melatonin added before, simultaneously or after OA treatment.

Microfilament organization was studied by RITC-phalloidin staining and fluorescence microscopy. Tau phosphorylation and tau levels were determined by Western Blot. The proteins were immunodetected by using a specific antibody that recognizes the tau´s 404 serine and the C-17 antibody that recognizes the tau COOH terminal sequence. The results showed that OA causes microfilament retraction toward the perinuclear region. The effect of OA was partially prevented by melatonin added 6h before simultaneously or after OA treatment. In addition, we found that melatonin added before, simultaneously or after OA treatment abolished tau hyperphosphorylation caused by OA. The results strongly suggest that melatonin acts as a neurocytoskeletal protector by decreasing tau hyperphoshorylation preserving the cytoskeletal structure. Data strongly suggest that melatonin may improve cognition by impeding neuronal damage by hyperphosphorylation and through establishing new neuronal pathways. 

## CONCLUDING REMARKS

Oxidative stress is a critical aspect of age-associated diseases, such as cancer, heart diseases and neurodegeneration. Most of free radical-related pathologies may have some another origin, but the common pathway once the process has begun, is oxidative damage. Since oxidative damage has the inherent capacity for perpetuating itself, free radical damage can be very severe.

Oxidant damage and mitochondrial dysfunction go together. This occurs under “normal” conditions in ageing, and under a variety of pathological conditions. Antioxidants, including melatonin should be tested for their efficacy in reducing the degenerative sign of aging as well as a protective agent against age-associated diseases that have an oxidative stress component.
